# Naturally Occurring and Artificial N9-Cytokinin Conjugates: From Synthesis to Biological Activity and Back

**DOI:** 10.3390/biom10060832

**Published:** 2020-05-29

**Authors:** Hana Vylíčilová, Magdaléna Bryksová, Vlasta Matušková, Karel Doležal, Lucie Plíhalová, Miroslav Strnad

**Affiliations:** 1 Department of Chemical Biology and Genetics, Centre of the Region Haná for Biotechnological and Agricultural Research, Faculty of Science, Palacký University, Šlechtitelů 27, CZ-783 71 Olomouc, Czech Republic; hana.vylicilova@upol.cz (H.V.); magdalena.bryksova@upol.cz (M.B.); vlasta.matuskova@upol.cz (V.M.); karel.dolezal@upol.cz (K.D.); 2 Laboratory of Growth Regulators, Palacký University & Institute of Experimental Botany ASCR, Šlechtitelů 27, CZ-783 71 Olomouc, Czech Republic; miroslav.strnad@upol.cz

**Keywords:** cytokinin sugar conjugates, glucoside, riboside, D-arabinoside, disaccharides, cytokinin nucleosides, plant biotechnology, *meta*-topolin, zeatin, plant tissue culture

## Abstract

Cytokinins and their sugar or non-sugar conjugates are very active growth-promoting factors in plants, although they occur at very low concentrations. These compounds have been identified in numerous plant species. This review predominantly focuses on 9-substituted adenine-based cytokinin conjugates, both artificial and endogenous, sugar and non-sugar, and their roles in plants. Acquired information about their biological activities, interconversions, and metabolism improves understanding of their mechanisms of action and functions in planta. Although a number of 9-substituted cytokinins occur endogenously, many have also been prepared in laboratories to facilitate the clarification of their physiological roles and the determination of their biological properties. Here, we chart advances in knowledge of 9-substituted cytokinin conjugates from their discovery to current understanding and reciprocal interactions between biological properties and associated structural motifs. Current organic chemistry enables preparation of derivatives with better biological properties, such as improved anti-senescence, strong cell division stimulation, shoot forming, or more persistent stress tolerance compared to endogenous or canonical cytokinins. Many artificial cytokinin conjugates stimulate higher mass production than naturally occurring cytokinins, improve rooting, or simply have high stability or bioavailability. Thus, knowledge of the biosynthesis, metabolism, and activity of 9-substituted cytokinins in various plant species extends the scope for exploiting both natural and artificially prepared cytokinins in plant biotechnology, tissue culture, and agriculture.

## 1. Introduction

**Figure 1:**
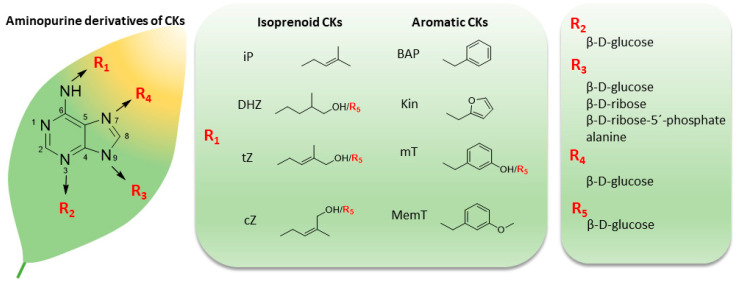
Structure of naturally occurring cytokinin (CK) aminopurine derivatives. The R_1_ determines the type of side chain, R_2_-R_5_ specify the type of CK conjugate.

Plants must adapt to continuous changes in their environments, such as variations in temperature, light, water status, nutrient availability, and pathogen attacks [1]. Many of these responses, and developmental processes, are controlled by interactions or ‘cross-talk’ between phytohormones (small organic signaling molecules) that include cytokinins (CKs), auxins, abscisic acid, gibberellins, ethylene, jasmonates, strigolactones, and brassinosteroids [2]. The first discovery of a CK (6-furfurylaminopurine, also known as kinetin, Kin), and its identification as a compound that strongly promotes cell division, in the mid-1950s [3,4], initiated intense investigations of CKs’ action mechanisms. This was mainly due to the obvious utility of CKs in tissue culture, and subsequently in plant biotechnology, agriculture, and horticulture [5,6]. We can distinguish two types of adenine-based CKs according to the substitution at N6 atom of adenine moiety. While isoprenoid CKs (IsCKs) are substituted by isoprenoid chain, aromatic CKs (ArCKs) by aromatic ring that can be further substituted by another functional group (Figure 1) [7].

Generally, CKs participate in control of cell growth and differentiation, and numerous physiological processes in plants. They increase antioxidant activity in plant tissues, which (inter alia) limits peroxidative damage of lipid membranes [8], and participate in chloroplast development [9], regulation of photosynthesis and senescence delay [8]. Other CK roles include participation in shoot and root growth [10], flowering [11], lateral bud formation [12], nitrogen accumulation in roots and translocation to leaves [13], carbohydrate supply [14], and responses to diverse environmental signals [15].

Numerous compounds with CK activity have been identified and structural requirements for CK activity have been formulated [16]. Naturally occurring CK free bases can be converted into the corresponding nucleosides, nucleotides, and glucosides. CKs also often occur as N9-alanine derivatives, but only free bases and ribosides seem to be biologically active [16]. Isoprenoid N6-isopentenyl aminopurine riboside (iPR) and *trans*-zeatin riboside (*t*ZR) are commonly present in plants and considered to be CK transport forms [17] while nucleotides are the key biosynthetic form. Conversion of CK free bases to their N-glucosides usually leads to their inactivation [18]. Moreover, 6-benzylaminopurine (BAP) and Kin, which are widely used in many commercial tissue culture techniques [19,20], due to their low cost and high efficacy, are probably the most well-known ArCKs [21]. Benzyalminopurine is used for the micropropagation of vast numbers of plant species [22,23,24,25,26,27]. Kin has been usually used in mixtures with α-naphthalene acetic acid (NAA) in tissue culture of many plants as well [28,29,30,31,32]. However, combinations of BAP and Kin in growth medium have often been used for micropropagation [33,34,35,36,37,38]. In addition, both Kin and BAP are more stable in vivo than naturally occurring IsCKs, which are more susceptible to fast degradation by CK oxidase/dehydrogenase, a key CK degrading enzyme [39]. Although BAP is currently the most affordable and widely used ArCK in tissue culture-based micropropagation, its utilization is associated with several disadvantages [40], mainly lateral root inhibition, growth heterogeneity, problematic acclimatization of plants in the greenhouse [41] and shoot tip necrosis [42]. Some authors attribute the inhibition of root initiation and growth to extensive accumulation of non-active CK N9-glucosides in the shoot base [43] or activation of ethylene production [44].

Hence, increasing research efforts have been geared toward enhancing the efficiency, and avoiding negative effects, of the commonly used CKs on root development. Generally, the easiest way to change the BAP properties is by a substitution on the benzyl ring [45,46]. However, CKs can also be substituted at several other positions of the purine ring, such as N1, C2, N3, N7, C8, and N9 [47]. All substitutions significantly influenced CK activity, but several N9-substituted CKs had no negative effects on root elongation, which was attributed to prevention of irreversible formation of 9-glucosides [48]. Here, we review current knowledge on O-, N7-, and N9-glucosides, L- and D-ribosides, D-arabinosides, deoxy-D-ribosides and other sugar CK conjugates. We also included some purely artificial mimetic derivatives, such as 9-tetrahydropyran-2-yl, 9-tetrahydrofuran-2-yl, 9-halogenalkyls, and other CK derivatives that are biologically active and could find potential applications in many important sectors, such as agriculture, tissue culture, the cosmetic industry, and medicine.

## 2. N7- and N9-Sugar Cytokinin Conjugates

### 2.1. Cytokinin 7- and 9-Glucosides

Cytokinins can form N-glucosides, in which glucose may be attached to the N3, N7, or N9 atoms of the purine moiety. CKs also form *O*-glucosides, in which glucose is linked via an oxygen atom bound to the benzene ring or N6-side chain attached to N6 atom of adenine-based CKs. *N*-glucosides are biologically stable and one of the most abundant naturally occurring CK forms. At certain circumstances, they may account for approximately 80% of the total CK content in plants [17]. Different glucose conjugates play different roles in CK transport, protection of CKs from degradation and reversible or irreversible CK inactivation [10]. Conjugation to the N3 atom has been described rather rarely. It has been assumed that both 7-glucoside and 9-glucoside formation is irreversible and inactivates CKs [49]. For example, it has long been known that BAP-9-glucoside (BAP9G) has weak activity in CK bioassays and does not release appreciable amounts of free active BAP [50]. Both aromatic and isoprenoid 9-glucosides have been synthesized via condensation of 6-chloropurine-9-glucoside with appropriate amines and found to be inactive in *Amaranthus*, tobacco callus, and senescence bioassays [51]. Moreover, tobacco callus grew more slowly on media supplemented with CK 9-glucosides than controls that received no CK treatment, and generally, 9-glucosylation dramatically reduced activities of all CKs tested in these assays [52]. The 7- and 9-glucosylation generally almost reduce CK activity completely in nearly all CK bioassays, including the radish cotyledon, *Amaranthus* betacyanin, oat leaf senescence, and tobacco pith callus bioassays [52]. This is because 7- and 9-conjugates are usually resistant to α-glucosidases, and thus cannot be hydrolytically converted into active CKs, unlike *O*-glucosides, which are conjugated via an oxygen atom [21]. Moreover, none of the *N*-glucosides tested reportedly triggered any response of *Arabidopsis thaliana* (L.) Heynh. CK receptors of *Arabidopsis* histidine kinase (CRE1/AHK4, AHK3) in a bacterial assay [53]. Very recently, distinct metabolisms of *N*-glucosides of N6-isopentenyladenine (iP) and *trans*-zeatin (*t*Z) were described. Despite of iP, both N9 and N7-*t*Z glucosides were cleaved to *t*Z free base [54]. Subsequently, constructed mathematical model provides estimation of the metabolic conversion rates. However, supplementary experiments using tritiated standards did not fully confirmed the findings. Therefore, in our opinion, because this study is in contradiction with many observations published before, it needs to be confirmed by detailed biochemical experiments before being fully accepted.

In the late 1980s, a novel zeatin-*O*-glucoside-9-glucoside was identified in young wheat spikes in [55]. This diglucoside was subsequently detected in transgenic *A. thaliana* plants overexpressing an *IPT* gene (encoding the key CK biosynthesis enzyme isopentenyl transferase) as dihydrozeatin-*O*-glucoside-9-glucoside [56]. A phosphorylated form of zeatin-9-glucoside was also identified. We can thus conclude that 9-glucosides are probably involved in homeostatic mechanisms that control endogenous CK levels, and biological activities of the mentioned forms in three CK bioassays are reportedly low. 

Natural formation of *N*-glucosides has attracted significant interest over many years, because it was considered to be a major barrier to the successful use of CKs in field applications [57]. Two enzymes that catalyze 7- and 9-glucopyranosylation of BAP were found in soluble extracts of expanded cotyledons of radish (*Raphanus sativus* L. cv. Long Scarlet) and purified more than 40 years ago [58]. In recent years, molecular approaches have been used to elucidate functions of various CK-specific glycosyltransferases and CKs have been shown to be deactivated by uridine diphosphate glycosyltransferases (UGTs) [59]. Uridine diphosphate glycosyltransferases, also called 1-glycosyltransferases, are the most common plant enzymes that catalyze transfers of sugar moieties from activated donor molecules to specific acceptor molecules such as phytohormones, secondary metabolites, and amino acids [60,61]. Two closely related *A. thaliana* genes encoding cytosolic enzymes with ability to catalyze CK *N*-glucosylation (UGT76C1 and UGT76C2) in vitro have been identified. Both recognize classical CKs such as *t*Z, dihydrozeatin (DHZ), BAP, iP or Kin, and glucosylate them mainly at the N7 and N9 atoms, but not N3 atom [57]. However, the 7-H tautomer is the favored state, so the N7 is most available for glucosylation by UGTs, and accordingly the two UGTs reportedly generate higher levels of 7-glucoside in vitro [57]. Subsequent experiments with transgenic plants confirmed that both glucosyltransferases can finely modulate CK responses via *N*-glucosylation, but UGT76C2 seems to have stronger effects [62,63].

### 2.2. Cytokinin 9-Ribosides

#### 2.2.1. IsCK 9-Ribosides

**Figure 2:**
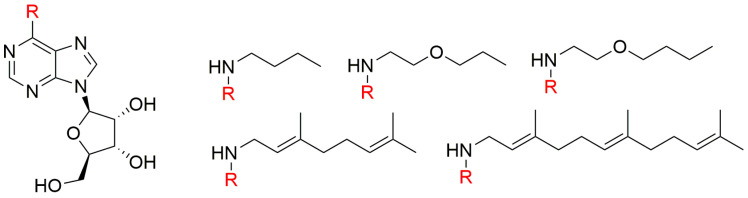
General structure of N6-substituted adenosines and their N6-substituents [68].

Isoprenoid CKs are ubiquitous in the plant kingdom [64] and regarded as the predominant type of CKs [65]. More than 50 years ago, 9-ribosides of Kin and iP were found to be 2- to 5-fold less active than their free bases in the tobacco callus assay [66], and effects of side alkyl chain hydroxylation on CKs’ growth-promoting activity in this bioassay were described [67]. Generally, the most striking effects observed are that hydroxylation of the isopentyl or isopentenyl chains at the 4-position increases this CK activity while hydroxylation at the 2- or 3- positions, reduces it. The same bioassay was also used to test a series of N6-substituted (N6-butyl-, N6-*N*-2-propoxylethyl-, N6-*n*-2-butoxyethyl-, N6-geranyl- and N6-farnesyl-) adenine ribosides (Figure 2). The N6-butyl and propoxyethyl adenosines showed CK activity, although they were less potent than *t*ZR. In contrast, the other compounds showed only marginal or none CK activity [68].

**Figure 3:**
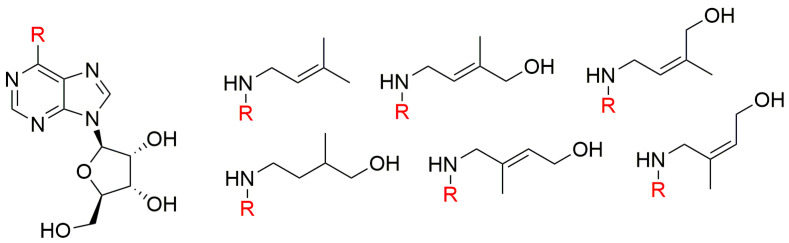
Compounds prepared and tested by Schmitz et al. in 1972 [69].

In tobacco bioassays, none of the geometric or position isomers of ZR and other compounds closely related to zeatin (Figure 3) was found to be more active than zeatin [69]. The 9-ribosyl derivatives of *t*Z, *cis*-zeatin (*c*Z), *trans*-isozeatin, and *cis*-isozeatin were also prepared and found to be less active than the original free bases [69].

Comparison of the cell-division stimulatory activity of iP and iPR in tobacco callus bioassay more than 40 years ago [70], and numerous subsequent experiments have shown that free bases generally have higher biological activity than corresponding ribosides [21]. Differences in relative activities could be explained by differences in the perception and transmission of the CK signals by various CK receptors. For example, the two *A. thaliana* CK receptors AHK3 and CRE/AHK4 are more sensitive to the IsCK bases *trans*-zeatin and iP than their ribosides, but AHK3 is more sensitive to ribosides than CRE1/AHK4 in vitro and the ability of *t*ZR to activate CRE1/AHK4 does not increase even after prolonged incubation [53]. Therefore, it was assumed that ribosides have genuine biological activity, with specificity for AHK3. Variations in ligand preference of three *Zea mays* L. histidine kinase receptors (ZmHK1, ZmHK2, and ZmHK3a) have also been detected, with indications that ZmHK2 is most sensitive to ribosides [71]. 

Comparison of the activities of *cis*-zeatin riboside (*c*ZR) and *t*ZR isomers and iP, has also shown that *t*ZR is more active than *c*ZR in stimulation of cucumber cotyledon expansion, retention of chlorophyll in detached leaf pieces, induction and stimulation of chlorophyll synthesis in cucumber cotyledons, and betacyanin synthesis in *Amaranthus caudatus* L. seedlings grown in the dark [72]. In addition, iP was less active than *t*ZR in all these bioassays, but more active than *c*ZR in the induction and stimulation of betacyanin and chlorophyll synthesis. Moreover, the ability of another IsCK, dihydrozeatin riboside (DHZR), to delay senescence of carnation flowers is similar to that of the free base [73].

Cytokinins are also synthesized by some phytopathogens to disrupt the hormonal balance and to facilitate niche establishment in their hosts. In pathology of *Rhodococus fascians* and related microorganisms, methylated CKs, have been repeatedly shown to play an important role [74,75,76]. Cytokinin ribosides can be methylated on side-chain or purine moiety. Moreover, 6-(4-hydroxy-1,3-dimethylbut-*trans*-2-enylamino)-9-β-D-ribofuranosylpurine (1-methylzeatin riboside), CK methylated on side chains, has been identified endogenously in *Pseunomonas syringae* pv *savastanoi.* Tests with the naturally occurring CK 1’-methylzeatin, its riboside and various derivatives have shown that they have stronger ability to stimulate synthesis of chlorophyll in etiolated cucumber cotyledons than *t*Z and *t*ZR, respectively [77]. In contrast, dihydro-4′-deoxy-1′-methylzeatin riboside proved to be inactive, mainly due to absence of the hydroxyl group at C4 of the side chain, and iPR was slightly active. Generally, the length of the alkyl side chain and *cis/trans* isomerism reportedly influence CK activity, and the presence of a hydroxyl group at the C4 atom seems to strongly promote it [77]. The same bioassay was used to test CK activity of *t*ZR and *c*ZR, and the ribosides were found to be less active than corresponding free bases [78]. Fas operon of *R. fascians* is essential for the enhanced production of CK mix including 2-methylthio derivatives of the zeatin ribosides, which are also important part of the pathogenicity mechanism [75,76].

Cytokinins are synthesized in many cell types, in both roots and shoots, and act both short and long distances [79]. Generally, *tZ*-type CKs, mainly *t*ZR, are transported from roots to shoots via xylem, whereas IsCKs are transported from shoots to roots via phloem [80]. While *t*Z is an active CK, *c*Z shows only limited CK activity [53]. In response to nitrogen availability, plants are thought to be able to modulate the relative ratio of *t*Z / *t*ZR in xylem sap and allows them to fine-tune the manner of shoot growth to adapt to changing environmental condition [81]. In addition, the ratio of *c*Z/*t*Z and their ribosides changes in behalf of *c*Z type needed for root hair elongation and phosphate allocation in the root during phosphate starvation [82]. Moreover, both *t*ZR and *c*ZR can reportedly suppress chlorophyll degradation in an oat leaf senescence assay and maize leaf segments in a drop bioassay but *t*ZR more effective than *c*ZR as well as in tobacco callus bioassay [64].

Cytokinin ribosides may also contain glucosyl conjugated via oxygen in the hydroxyl group of the side chain of IsCKs [83]. These CK-riboside-*O*-glucosides, namely *trans*-zeatin riboside-*O*-glucoside (*t*ZROG), *cis*-zeatin riboside-*O*-glucoside (*c*ZROG), dihydrozeatin riboside-*O*-glucoside (DHZROG), and the corresponding o-glucosides of free bases, are endogenous CKs that have been recorded in many species of vascular plants [84], for example *Nicotiana rustica* L. [85], *Vinca rosea* L. [86], *Populus alba* L. [87], and *Tulbaghia* L. [88]. They have also been detected in non-vascular plants, particularly in the moss *Physcomitrella patens* (Hedw.) Bruch and Schimp., in which analysis of CK profiles revealed that *c*ZROG and *t*ZROG were the most abundant intracellular conjugates of CKs [89]. Generally, *O*-glucosides of zeatin-type CKs are considered important for storage and transport because they are resistant to CK oxidase/dehydrogenase-mediated breakdown, and easily converted into the active form by the action of β-glucosidases [90]. Moreover, findings that *t*ZROG is biologically active in an *A. thaliana* reporter gene test but does not trigger responses by either CRE1/AHK4 or AHK3 receptors of *A. thaliana* [53], could be due to rapid breakdown of this metabolite, yielding biologically active free base in *A. thaliana*. Evaluations of endogenous CKs’ distributions indicate that *O*-glucosides accumulate most strongly in roots [88,91].

#### 2.2.2. ArCK 9-Ribosides

Neither ArCK ribosides nor free ArCKs were identified as naturally occurring compounds for many years after the discovery of CKs in plants, although many were prepared in the laboratory and used widely in tissue culture almost immediately after their discovery. Their natural origin was only confirmed with the reported isolation of 6-(2-hydroxybenzylamino)-9-β-d-ribofuranosylpurine (*ortho*-topolin riboside, *o*TR) from poplar leaves in 1975 [92] and from *Zantedeschia aethiopica* (L.) Spreng. fruits in 1980 [93]. Kinetin riboside (KinR) was initially identified as a naturally occurring conjugate in coconut water [94] and BAP 9-β-ribofuranoside (BAPR) has been identified in natural plant (*Cocos nucifera* L.) material [95]. Benzylaminopurine 9-β-ribofuranoside is reportedly more active than zeatin-9-riboside (*t*ZR) in the tobacco callus bioassay, both BAPR and *t*ZR have high activity in the *Amaranthus* bioassay (but lower than that of the corresponding free bases), and BAPR has weaker anti-senescence activity than *t*ZR [51]. Benzylaminopurine 9-β-ribofuranoside is also a putative precursor of hydroxybenzylaminopurines (topolins) in plant tissues, and hydroxylation of the benzyl ring at *meta-* and *ortho*- positions, yielding *meta*-topolin-9-riboside (*m*TR) and *o*TR, putatively promote CK activity and/or deactivate BAPR [96]. Thus, for example, *m*TR and *o*TR reportedly have higher and lower activity in CK bioassays than corresponding free bases *meta*-topolin (*m*T) and *ortho*-topolin (*o*T), respectively [51]. Since their discovery, highly active *m*T and its riboside have been employed for culture initiation, protocol optimization and for counteracting various in vitro induced physiological disorders in many species. Evidence from various studies indicate the rising popularity and advantages (although not universal for all species) of topolins compared to other CKs [97]. For example, adding *m*TR to the culture medium during in vitro propagation of potato can significantly improve survival rates [98]. Further, treatments including *m*TR provision can overcome the problematic occurrence of necrotic shoot-tips associated with use of BAPR and its free base in micropropagated *Harpagophytum procumbens* (Burch.) DC. ex Meisn. [42]. Moreover, in vitro regeneration rates of explants of the orchid *Ansellia africana* Lindl. are significantly higher in *m*TR-containing media than in other tested media [99], and it has proposed utility as an alternative to other commonly used CKs in micropropagation of smoke bush (*Cotinus coggygria* Scop.) [100]. However, it should be noted that there are plant species that respond better to other CKs than topolins; hence topolins should not be taken as a panacea and must pass through the routine process of selection and optimization of tissue culture protocol [97].

It should be emphasized that the hydroxyl group on the benzyl ring in *m*T allows reversible *O*-glucosylation. Before or after *O*-glucosylation, the N9 position can be conjugated with ribose, forming *meta*-topolin riboside-*O*-glucoside (*m*TROG), which has been detected as a main metabolite of *m*T in all parts of micropropagated *Spathiphyllum floribundum* (Linden & André) N.E.Br. However, *m*TROG can be easily cleaved in plant tissues by β-glucosidases, and thus it penetrates plant tissue with biologically active *m*T or its riboside. On the other hand, major metabolite of widely used BAP is the highly stable and biologically inactive BAP9G, which accumulates in plant basal parts and might be responsible for undesirable inhibition of root development. Plants treated with *m*T reportedly produce significantly more, and longer, roots than counterparts treated with BAP during acclimatization [40]. Clearly, the presence of a hydroxyl group gives topolins a structural advantage over BAP, since it allows formation of O-glucosides, which cannot be formed from BAP [5].

Recently, two endogenous ArCK isomers of topolins, *ortho*-topolin-9-riboside-*O*-glucoside (*o*TROG), and *meta*-topolin-9-riboside-*O*-glucoside (*m*TROG) were detected in microalgae [101]. Roughly concurrently, two *O*-glucosides *m*TROG and *para*-topolin-9-riboside-*O*-glucoside (*p*TROG) were detected in shoots of tissue-cultured *Aloe polyphylla* Pillans plants treated with BAP, at levels that depended on the type of gelling agent used to solidify the medium [102].

Targeted searches for naturally occurring ArCKs in *A. thaliana* plants and *Populus x canadensis* Moench cv. Robusta leaves led to the identification of two methoxy ArCK ribosides: 6-(2-methoxybenzylamino)purine-9-riboside (*ortho*-methoxytopolin riboside) and 6-(3-methoxybenzylamino)purine (*meta*-methoxytopolin-9-riboside, Me*m*TR). In the same study, these compounds were found to have higher CK activity in tobacco callus, *Amaranthu*s, and detached wheat leaf senescence bioassays than BAP and *t*Z [103]. Recently, Me*m*TR was also found to have stronger anti-senescence effects during early senescence than BAP in micropropagation of rose [104]. The high potential utility of *m*TR and Me*m*TR was subsequently studied to replace BAP and zeatin in micropropagation of *A. polyphylla* [105]. Additionally, Me*m*TR has shown high potential for promoting adventitious shoot production in micropropagation of the endangered endemic shrub *Barleria greenii* M. Balkwill and K. Balkwill [106]. In further recent studies of the effects of *m*T, *m*TR, *meta*-methoxytopolin (Me*m*T), and Me*m*TR, micropropagated banana plantlets regenerated with Me*m*TR had significantly longer roots and higher shoot/root ratios than controls and BAP-treated plants. Me*m*TR and *m*TR also induced higher chlorophyll a/b ratios than BAP treatments, which were closer to the optimum for photosynthesis during acclimatization [107].

**Figure 4:**
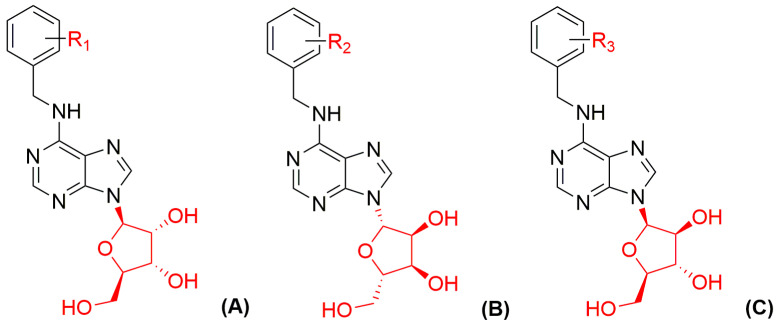
General structures of aromatic (**A**) 6-benzylaminopurine-9-β-D-riboside derivatives (R_1_ = X, CH_3_, OCH_3_, OH, OCHF_2_, OCF_3_, CF_3_ or a combination of these groups), (**B**) 6-benzylaminopurine-9-β-L-riboside derivatives (R_2_ = H, F, Cl, OCH_3_ or OH) and (**C**) 6-benzylaminopurine-9-β-D-arabinoside derivatives (R_3_ = X, CH_3_, OCH_3_, OH, OCF_3_, CF_3_ or NH_2_).

Based on some of the findings described above, numerous BAPR analogues with various substituents on the benzyl ring (Figure 4) were synthesized and their biological activities were studied [45]. The results suggested that position-specific steric effects of the benzyl ring substituents decrease CK activity, with strength declining in the following order: *meta* > *ortho* > *para* [51]. The highest activities were observed in the wheat leaf senescence bioassay (WLS), in which some compounds delayed senescence up to 2.2 times more efficiently than BAP, and almost 50% of the prepared compounds were more active than BAP. It was assumed that substituents with high electronegativity enhance the activity of aromatic ribosides, probably through hydrogen bond formation with electron donors of a CK receptor [108]. This assumption was supported by the findings that fluoro derivatives are the most active compounds [45]. Important variations in the selectivity of disubstituted derivatives were also reported. For example, 6-(2,4-dichlorobenzylamino)purine-9-riboside was active in the tobacco callus bioassay, but not in other CK bioassays, while 6-(3,4-dichlorobenzylamino)purine-9-riboside was active in WLS and *Amaranthus* assays. Therefore, small changes in benzyl ring substitution can clearly lead to significant changes in specificity of compound biological activity [45]. Interestingly, none of the prepared BAPR derivatives significantly activated either of the *A. thaliana* CRE1/AHK4 or AHK3 CK receptors [45]. Thus, it was assumed that their biological activities involve other mechanisms. Furthermore, two of these compounds, 6-(2-hydroxy-3-methoxybenzylamino)purine-9-β-D-ribofuranoside and 6-(2,4-dimethoxybenzylamino)purine-9-β-D-ribofuranoside, were isolated from *A. thaliana* and *Agrobacterium tumefaciens* extracts, and identified as new plant growth substances [45].

Another derivative, 6-(3-fluorobenzylamino)purine-9-riboside (FBAPR), was found to promote shoot multiplication significantly more strongly than BAP in rose micropropagation [104]. Similarly, FBAPR treatment resulted in formation of significantly more, but smaller, new shoots during in vitro cloning of *Phalaenopsis amabilis* (L.) Blume hybrids (which is generally characterized by slow growth and low multiplication rates), than treatment with either 6-(3-fluorobenzylamino)purine (FBAP) or BAP [109]. The results suggested that use of fluorinated BAPRs could substantially improve in vitro micropropagation of *P. amabilis* [109].

In our opinion, there is enough evidence to conclude that 6-benzylaminopurine-9-β-D-ribosides, bearing appropriate substituent on the phenyl ring, have a great potential to be a solution to many problems afflicting current tissue culture industry and agriculture in general (such as drought and other abiotic stress tolerance).

**Figure 5:**
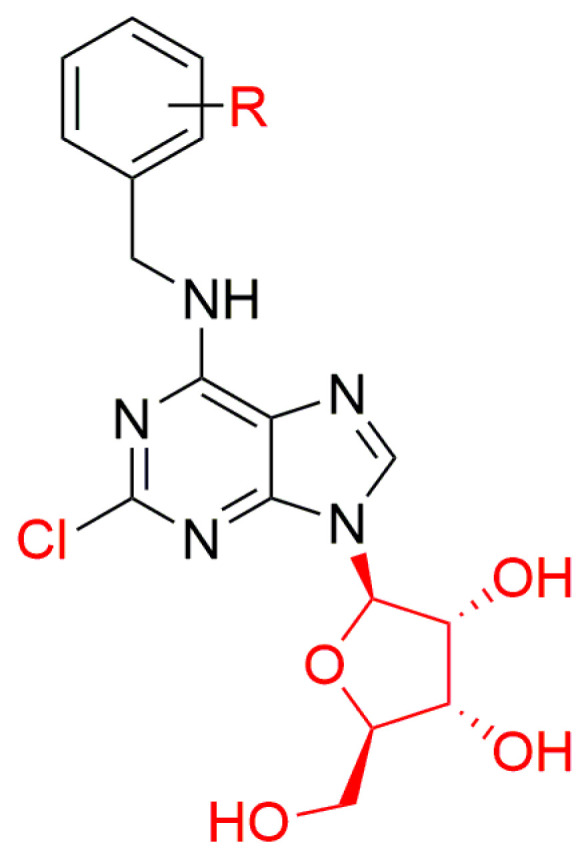
General structure of 2-chloro-6-disubstituted benzylaminopurine riboside derivatives, where R = halogens [46].

Recently, the number of available N9-conjugates of ArCK sugars with halogen atoms on benzyl ring has been extended by the preparation of new aromatic 2-chloro-6-(halogenobenzylamino)purine ribosides and their biological activity was studied [46]. A group of 2,6-disubstituted CK derivatives was also prepared by reacting 2,6-dichloropurine riboside with the appropriate benzylamines in the presence of triethylamine in *n*-propanol [45] and their structures are shown in Figure 5.

Derivatives bearing a fluorine atom on the benzyl ring have generally strong activity in the WLS bioassay; 2-chloro-6-(4-fluorobenzylamino)purine-9-riboside, the most potent compound, delayed loss of 50% chlorophyll 1.96-fold longer than BAP [45,46]. The most active compounds are always found among the derivatives bearing a halogen in the *meta* or *para* position of the N6-benzyl ring. Moreover, high-throughput comparative gene expression analysis revealed that two tested halogenated ArCK derivatives upregulated several genes associated with photosystems I and II, as well as other components of the photosynthetic apparatus. Both compounds delayed the onset of senescence by maintaining chlorophyll and carotenoid levels and increasing the relative abundance of light harvesting complex II, thereby protecting photosystem II activity. Prepared compounds showed similar biological activity to standard BAP in tobacco callus and A*maranthus* bioassays. Most of the derivatives did not trigger CK signaling via the AHK3 and CRE1/AHK4 receptors from *A. thaliana*, but some of them specifically activated the ZmHK1 receptor from *Zea mays* and were more active than BAP in the ARR5::GUS CK bioassay using transgenic *A. thaliana* plants [46]. 

It should be noted that halogenated ArCK ribosides can induce CK responses that could be caused by their conversion to the free bases [21,46]. There may also be a different sensing mechanism for ArCKs in plants [45] and there is strong evidence of the presence of another extracellular CK perception system involving plasma-membrane-bound receptors [110].

Recently, several derivatives of 6-benzylamino-9-β-L-ribofuranosylpurines were synthesized (Figure 4) and their CK activities were measured [111]. These were prepared by one-step nucleophilic substitution, starting with reaction of β-L-inosine with corresponding benzyl amines in the presence of Castro reagent and Hünig base, largely following previously published procedures [112]. CK activity of the newly prepared derivatives was tested in *Amaranthus*, tobacco callus, and WLS bioassays. Generally, the L-enantiomers had significantly weaker biological activity in WLS bioassays than corresponding D-enantiomers [111]. For example, classical *meta*-topolin-9-β-D-riboside (D-*m*TR) had 2.37-fold higher and its L-enantiomer had 3.44-fold lower activity than BAP, respectively. The D-ribosides were also significantly more active in the tobacco callus bioassay [45].

**Figure 6:**
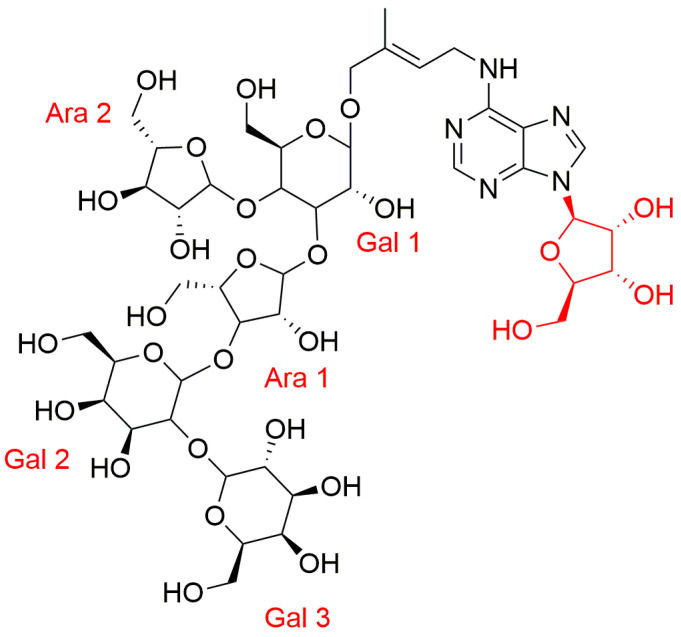
Structure of 14-*O*-{3-*O*-[β-D-galactopyranosyl-(1→2)-α-D-galactopyranosyl-(1→3)-α- L-arabinofuranosyl]-4-*O*-(α-L-arabinofuranosyl)-β-D-galactopyranosyl}-trans-zeatin riboside (G_3_A_2_-ZR) [113].

A remarkable compound, detected in coconut milk, was 14-*O*-{3-*O*-[*β*-D-galactopyranosyl-(1→2)-*α*-D-galactopyranosyl-(1→3)-*α-*L-arabinofuranosyl]-4-*O*-(*α*-L-arabinofuranosyl)-*β*-D-galactopyranosyl}-*trans*-zeatin riboside (G_3_A_2_-ZR) (Figure 6). The discoverers found that at least 20% of the CK activity of coconut milk could be attributed to G_3_A_2_-ZR [113]. Thus, G_3_A_2_-ZR is an order of magnitude more potent than 1,3-diphenylurea and an order of magnitude less potent than *t*ZR. Its CK activity in tobacco callus could be mediated by hydrolysis to zeatin and, in addition, this conjugate could be preferentially accepted because it is water soluble, while zeatin and ZR are more lipophilic and have lower solubility in water. Production of a highly water-soluble CK (or precursor) such as G_3_A_2_-ZR and its accumulation in coconut milk could be beneficial for nourishment of the immature coconut embryo [113].

Cytokinin ribosides and riboside monophosphates (ribotides) were commonly reported as metabolites of exogenously applied CKs, and their interconversion was demonstrated by radiolabeling in a study of lettuce seed germination. The results showed that exogenously applied [^14^C]Kin is rapidly metabolized in lettuce seeds to the corresponding nucleoside and nucleotide [114,115]. Another endogenous ArCK ribotide (BAPR-5′-monophosphate - BAPRMP) and isoprenoid CKs (isopentenyladenosine-5′-monophosphate, dihydrozeatin- riboside-5′-monophosphate, and zeatin riboside-5′-monophosphate) have been found in aerial parts of the coconut palm [95]. In tests of *trans*-zeatin riboside-5′-monophosphate in CK receptor bacterial assays, it activated the CRE1/AHK4 but not the AHK3 receptor. The ribotide was also active in the ARR5::GUS CK bioassay. Recently described BAPRMP derivatives have potential medical uses because they have anticancer, antimitotic, and pro-apoptotic activities in animal and human cells [116]. Furthermore, a group of BAPR-5′-*O*-di- and tri-phosphate derivatives have similar activities against selected cell lines to the parent ribosides [117]. The activity of such ribotides has also been recently patented [116].

### 2.3. Purine 9-(2′-Deoxyribosides) Cytokinin Conjugates

**Figure 7:**
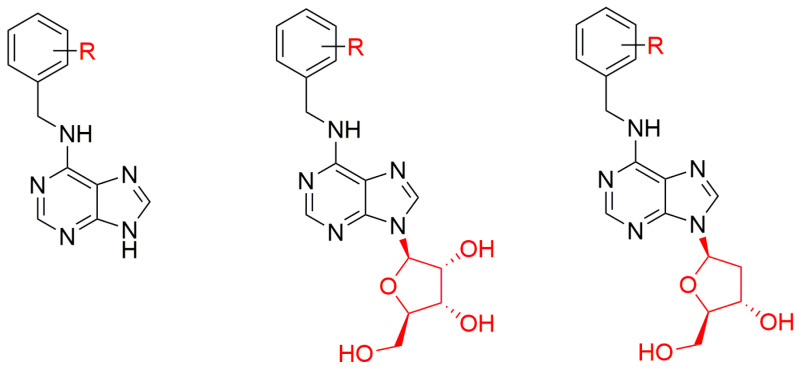
Comparison and general structures of free base, riboside and 2′-deoxyriboside derivatives, where R = X, OH, OCH_3_, CH_3_, OCF_3_, CF_3_ or a combination of these groups.

Purines substituted at N9 atom with 2′-deoxyribose are important components of various biomolecules that are essential for physiological processes, e.g. DNA, and various signaling molecules [118]. Test results ca. 30 years ago showed that zeatin-9-(2′-deoxyriboside) (*t*Z2′dR) and its monoacetyl and triacetyl derivatives were able to stimulate chlorophyll synthesis in etiolated cucumber cotyledons but very weakly [77]. On the other hand, *t*Z2′dR inhibited the DNA-polymerizing reaction catalyzed by DNA-polymerase I of *Escherichia coli* [119]. In addition, *cis*-zeatin-2′-deoxyriboside reportedly had no CK activity in the tobacco callus bioassay [78]. Recently, benzyl ring-substituted 6-benzylamino-9-(2′-deoxy-*β*-d-ribofuranosyl)purine derivatives (Figure 7) have been prepared [112,120] and tested in various classical CK bioassays. The results showed that attachment of a 2′-deoxyribosyl moiety to the N9 atom significantly enhanced the prepared derivatives anti-senescence activity in the WLS bioassay, relative to activities of both corresponding free bases and ribosides [120]. 

In the *Amaranthus* bioassay, replacement of ribose by a 2′-deoxyribose sugar moiety did not significantly affect activity of most of the prepared derivatives, but the activity of some of them reached the maximum level at ca. 10-fold higher concentration (100 µM) than BAP [120]. Furthermore, 6-(3-hydroxybenzylamino)-9-(2′-deoxyribofuranosyl)purine and 6-(3-methoxybenzylamino)-9-(2′-deoxyribofuranosyl)purine reportedly have significantly higher anti-senescence and chlorophyll maintenance activities than BAP in WLS assays. In contrast, most tested compounds had lower activity than BAP in tobacco callus bioassay [120].

Purine 2′deoxy-nucleoside analogues have been reported to have antiviral potency. Benzylaminopurine 9-β-ribofuranoside and N6-benzyl-2′-deoxyadenosine are active against alphaviruses (Semliki Forest and Sindbis viruses) [121] and Human enterovirus 71 [122,123]. 

Generally, despite the fact that 2′-deoxyadenosines do not bind the CK receptor, they possess an incredible anti-senescent activity in plant bioassays [120]. Thus, a simple synthetic exchange of the pentose sugar group on the N9 atom led to the preparation of substances, which are no longer apparently CKs, but which have a high added value due to the preservation (and improvement) of influencing leaf senescence. 

### 2.4. Purine N9-Arabinosides and Their Precursors

More than 40 years ago, a small library of iP analogues substituted at N9 by a ribose or arabinose with the side chain containing acetylenic, dimethylaminoethyl, pyridylmethyl, cyclopropylbenzyl, or cyclopropylmethyl functional groups was synthesized and tested for CK activity in the tobacco callus bioassay [124]. Most of them showed moderate or strong activity. Replacement of D-ribose by D-arabinose or replacement of the isopentenyl side chain also lowered CK activity [124].

A group of 6-alkylaminopurine arabinosides was also prepared in the 1980s [125,126,127,128], by transferring the arabinosyl moiety from a pyrimidine arabinoside to the purine aglycone [129]. These compounds were found to be selectively active against varicella-zoster virus [130]. This was not surprising because the β-anomer of 9-(D-arabinofuranosyl)adenine (Ara-A), and a series of N6- or C8-substituted variants of Ara-A had been previously synthesized and found to have in vitro antiviral activities against herpes simplex and vaccinia viruses as well [131]. Some other derivatives 8-amino-9-(β-D-arabinofuranosyl)adenine and 8,5′-anhydro-8-oxy-9-(β-D-arabinofuranosyl)adenine were also tested against vaccinium and herpes simplex [132,133]. However, the results showed that the substitution of Ara-A’s C8-atom caused loss of antiviral activity against both tested viruses. Finally, the tested derivatives carrying substituents at the N6 atom of the adenine moiety also had lower antiviral activity than their parent compounds, except for N6-(β-naphthylmethyl)-Ara-A [131].

Recently, a new class of non-toxic CK 9-(β-D-arabinosides) (Figure 4) was prepared according to a previously published protocol with a slight modification [112]. It is based on by reaction 9-(β-D-arabinofuranosyl)hypoxanthine with the corresponding benzylamines in the presence of Castro′s reagent and Hünig′s base [134]. In the *Amaranthus* bioassay, none of the derivatives had stronger activity than BAP, and, in addition, they exhibited low or modest activity (6–40% of BAP activity) also in the callus bioassay. These data suggested that the CK 9-(β-D-arabinosides) have only weak CK activity. However, it is interesting to note that several of the new derivatives had similar or higher activity in the WLS bioassay than BAP. These findings indicate that the new compounds can specifically affect the physiological processes associated with senescence and/or stress without being active CKs in receptor assays. Metabolic conversion of 6-benzyladenine arabinoside (BAPA) appears to be similar to that shown by BAP and is related to the formation of inactive CK 7- and 9-glucosides that are responsible for the aberrant root formation after BAP treatment [107].

### 2.5. Cytokinin Disaccharide Conjugates

**Figure 8:**
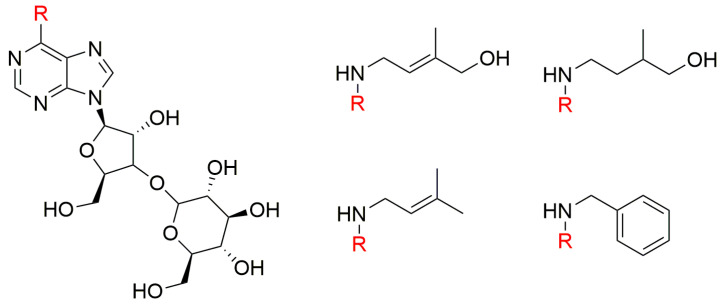
CK disaccharides (Z9RG, DHZ9RG, iP9RG) reported by Zhang et al. (2001) and BAP9RG reported by Auer and Cohen (1993) [138,140].

In the early 1980s, a novel isoprenoid conjugate of *t*ZR with a hexose moiety was identified by analyses of MS spectra of *Pinus radiata* D. Don bud extracts, indicating that the hexose moiety, attached to the ribose moiety, was probably glucose [135]. The zeatin disaccharide conjugate was active in a soybean hypocotyl bioassay [135], and subsequently detected in buds of the conifer Douglas fir [136]. Structures of three novel endogenous CK ribosyl-linked glycosides— dihydrozeatin-9-glucopyranosyl riboside (DHZ9RG), 6-(2-isopentenylamino)purine-9-glucopyranosyl riboside (iP9RG) and *trans*-zeatin-9-glucopyranosyl riboside (*t*Z9RG)—were identified (Figure 8) some years later, together with their phosphorylated forms, in mature buds of *P. radiata* [137]. The cited authors suggested that synthesis of these CK glycosides and their phosphorylated forms in conifers must involve enzymes that do not participate in formation of glucosides and nucleotides of traditional CKs [137]. Moreover, levels of phosphorylated CK disaccharides (*t*Z9RG and DHZ9RG) in *P. radiata* positively correlated with numbers of fascicle needle primordia in the adult buds [138]. Disaccharides have been found to be the major BAP metabolites formed during organogenesis in *Gerbera jamesonii* Bolus [139] and were detected in *Petunia hybrida* Vilm. [140].

In both of these cases, the culture media were supplemented with BAP, so it appeared that in the angiosperms new glycosides were synthesized from the aglycone present in the medium [137]. Moreover, 6-benzylaminopurine-9-glucopyranosyl riboside (BAP9RG, Figure 8) may be an important component of the metabolic regulation of the pool of active CKs, which is responsible for shoot organogenesis in culture [140]; it was also recently identified in tissues of the conifer *Pinus pinea* L. during adventitious bud formation in vitro after BAP treatment [141].

The phosphorylated form of BAP9RG was subsequently identified in metabolic profiling of mature *P. radiata* bud fragments cultured on BAP containing medium. In conclusion, BAP induces reinvigoration of the mature buds, in which BAP is converted into metabolites including BAP9RG and 6-benzylaminopurine-9-glucopyranosyl riboside phosphate (BAP9RGP). Anatomical examinations revealed that BAP inhibited development of secondary needle primordia and the reactivated meristem regained the ability to produce green primary needles with juvenile characteristics [142]. Understanding maturation of *P. radiata*, and other trees, is very important in clonal forestry, i.e., production of genetically identical trees from the same parental material [138,143]. Due to the frequent isolation of these disaccharides in conifers, it seems that these disaccharides have an irreplaceable function in their development, which is related to further improvement that might follow in tissue culture of tree species.

## 3. Non-Sugar N9-Substituted Cytokinins

A number of non-sugar 9-substituted derivatives of CKs have been described, several of which occur naturally and were discovered in plant tissues, such as 9-alanyl derivatives [7]. Most non-sugar 9-substituted CKs have been prepared as mimetics of CK sugar conjugates in the laboratory conditions [66,144,145,146,147]. We summarize current knowledge of these non-sugar 9-substituted CK derivatives and their biological activity in the following sections.

### 3.1. 9-Alanyl Derivatives

Attachment of the amino acid alanine to the N9 atom of the purine moiety in zeatin results in formation of 9-alanylzeatin and 9-alanyldihydrozeatin. These two naturally occurring isoprenoid CKs were named lupinic and dihydrolupinic acid, respectively, because they were initially identified in *Lupinus angustifolius* L. seeds [148]. Later, a novel transferase, which catalyzes conversion of zeatin to lupinic acid, was isolated and purified from *Lupinus luteus* L. cv. Weiko III seeds [19,149]. Lupinic acid is metabolically stable, but inactive or much less active than zeatin in CK bioassays, such as tobacco callus and radish cotyledon assay [149,150]. In contrast, lupinic acid has appreciable activity in soybean callus and *Amaranthus* bioassays [150], so its apparent CK activity acid depends on the assay. Release of free zeatin from lupinic acid has been observed, indicating that alanine conjugates may serve as potential storage rather than deactivation forms [151].

### 3.2. Synthetic 9-Substituted Alkyl, Cycloalkyl, and Halogenoalkyl CK Derivatives

**Figure 9:**
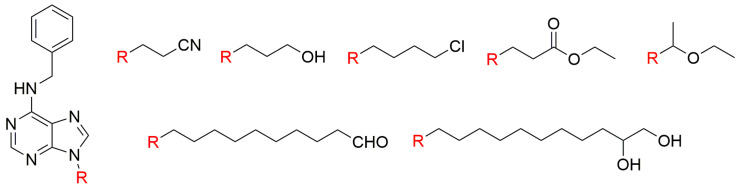
Structures of aliphatic chains substituted at the N9 atom of BAP [145].

Substitution at the purine N9 atom with alkyl or cycloalkyl groups significantly affects CK activity. Almost 50 years ago, it was reported that 9-methoxymethyl, 9-propyl, and 9-cyclohexyl derivatives of BAP (Figure 9) are less active than the free base in tobacco and soybean bioassays [144]. Several 9-substituted halogenoalkyl derivatives of BAP were also tested in the soybean senescence assay. The most potent, 9-(4-chlorobutyl), had slightly more ability than BAP to promote chlorophyll retention in intact soybean leaves. Its activity is probably due to easy dealkylation and release of free BAP, but in general, the mode of action of such 9-alkyl CK remains unclear [145].

**Figure 10:**
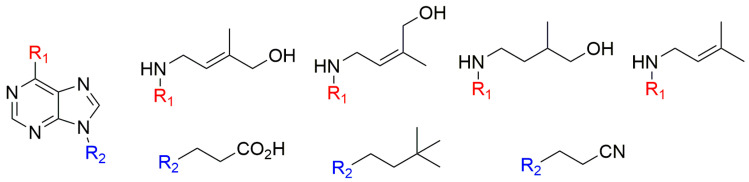
Structures of compounds prepared by Corse et al. (1989). On N6 (R1): tZ, cZ, DHZ, and iP side chains. On N9 (R2): 2-carboxyethyl, 2-carbo-*t*-butoxyethyl and 2-nitriloethyl) [146].

Subsequently, series of 9-substituted ethyl derivatives of four naturally occurring CKs (*t*Z, *c*Z, DHZ, iP Figure 10) were synthesized [146]. All of these derivatives were less active than the parent CKs in the soybean callus bioassay and their relative activities were more dependent on the structure of the isoprenoid side chain. The results clearly showed the importance of the structure and size of N9 substitutions and indicated that steric effects cause much lower losses of biological activity than the polarity of the substituent functional groups [146].

**Figure 11:**
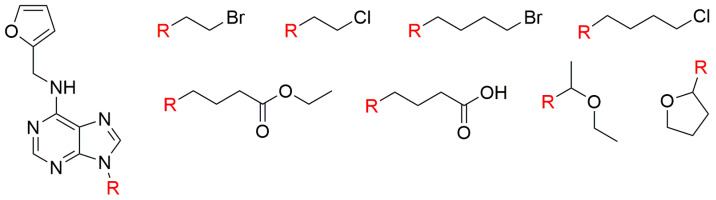
Structures of Kin derivatives prepared by Mik et al. (2011) [157].

Early experiments showed that many CKs (for example, Kin, *t*Z, and BAP) can delay senescence in detached leaves of various plant species, such as wheat, soybean [145,152], barley [153], rice [154], and oat [155]. These studies also showed that CKs can prolong the lifespan of cut carnation flowers [156]. Many CK derivatives have been prepared and tested, including various alkyls, halogenoalkyls and cycloalkyls, inter alia a group of 9-substituted Kin derivatives with halogenoalkyl, aliphatic or cyclic ether, and carboxylic chains (Figure 11) [157]. Derivatives substituted at the N9 atom with a short halogenoalkyl (chloroethyl, bromoethyl) have higher activity in tobacco callus bioassay than Kin, and derivatives with an aliphatic group and/or cyclic ether at the N9 atom have significant activity in this assay [157].

In the *Amaranthus* bioassay, these halogenoalkyl Kin derivatives were found to be only slightly active. Generally, the aliphatic and cyclic ethers were the most active, followed by halogenoethyl and halogenobutyl derivatives, while substances bearing 9-carboxylic chains were totally inactive. Halogenoalkyl derivatives also significantly delayed senescence, and their activity seems to depend on both the length of the alkyl chain and the halogen atom. CKs with short N9-halogenoalkyls (chloroethyl or bromoethyl) had the highest activity [157].

**Figure 12:**
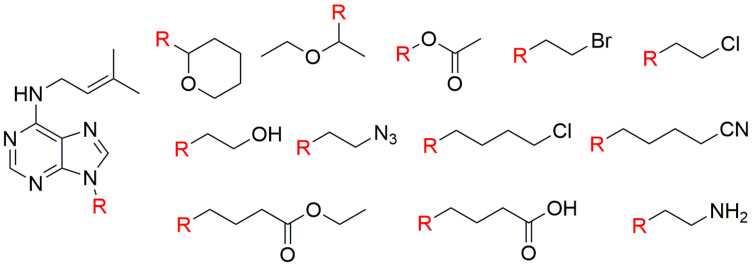
Structures of iP derivatives prepared by Mik et al. 2011 [158].

Subsequently, a series of iP derivatives specifically substituted at the purine N9 atom by ethoxyethyl and C2-C4 alkyl chains terminated by various functional groups (Figure 12) were prepared [158]. These compounds were synthesized using a previously described procedure [159] with slight modification. 

Substitution at the purine N9 atom with ethoxyethyl-, acetoxy-, azido-, 4-chlorobutyl-, and 3-cyanopropyl groups significantly improved iP cell-proliferation activity in tobacco callus bioassay. Generally, most of the derivatives showed high activity in the *Amaranthus* bioassay. However, the prepared derivatives did not show antisenescence activity in the WLS assay [157], probably because iP itself has much weaker activity in this bioassay than other CKs, such as BAP, Kin, and *t*Z [158].

**Figure 13:**
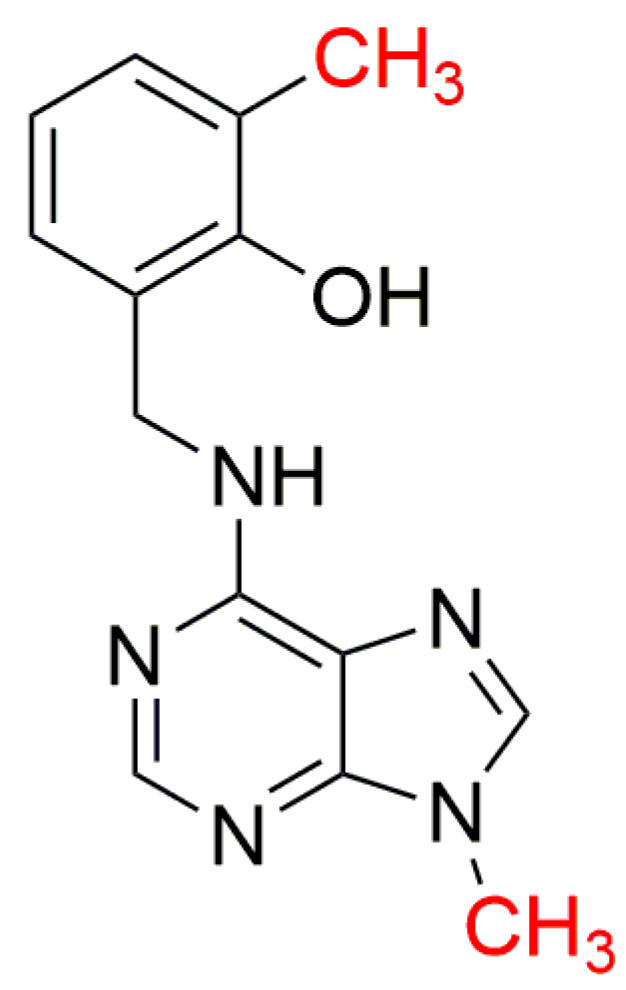
Structure of N9-methylated PI-55 [160].

N9-methylated CK antagonist 6-(2-hydroxy-3-methylbenzylamino)purine (PI-55, Figure 13), has also been synthesized and tested in CK bioassays [160]. PI-55 was the first identified CK receptor antagonist. This ‘anti-cytokinin’ has strong inhibitory effects on CK-induced responses in various bioassays, but also accelerates germination of *A. thaliana* seeds and promotes both root growth and formation of lateral roots [161]. However, methylation of the N9 atom caused complete loss of CK activity in all of three CK bioassays used to test them [160].

It seems that alkyl substituent at N9 atom does not have exclusive effect on CK activity itself. It is rather the combination of appropriate 9-alkyl and functional 6-substitution of adenine, often containing hydrocarbon residue with suitably located oxo or hydroxyl groups. 

### 3.3. 9-(Tetrahydropyran-2-yl) and 9-(Tetrahydrofuran-2-yl)ated CKs

In a study reported in 1967, 6-benzylamino-9-(tetrahydropyran-2-yl)purine (BAP9THP) was prepared and found to promote chlorophyll retention (and senescence delay) in plant tissues exceptionally strongly, and growth of tobacco callus almost as strongly as BAP. Its high activity was attributed to the lability of the 9-substituent [66]. Other early studies showed that some synthetic BAP9THP derivatives could stimulate tiller bud elongation in cereals [162] and increase numbers of apple and grape fruits [163,164]. A comparative study published in 1981 demonstrated that activities of BAP and various 9-substituted derivatives in the promotion of lettuce seed germination declined in the following order: BAP = 9-BAP9THP > 9-methyl BAP > 9-methoxymethyl BAP > 9-cyclopentyl BAP > 9-cyclohexyl BAP [115]. Later, 6-benzylamino-9-(tetrahydrofuran-2-yl)purine (BAP9THF) was prepared, its impact on leaf senescence was studied, and both BAP9THP and BAP9THF were found to delay senescence and induce several growth responses more strongly than BAP. The increased senescence-retarding activity of these compounds was at least partially attributed to the gradual cleavage of pyranyl or furanyl and release of free base there form [145]. 6-benzylaminopurine and BAP9THP have been reported to induce adventitious shoot formation significantly more strongly than iP or Kin [165]. Generally, 2-tetrahydropyranyl and 2-tetrahydrofuranyl cyclic ether groups are widely used in organic chemistry as protective groups and can be easily removed in acidic conditions [166]. The 9THP- or 9THF-substituted Kin and other 9THP and 9THF ArCKs have significant anti-senescence effects, as previously described for BAP [115]. 

**Figure 14:**
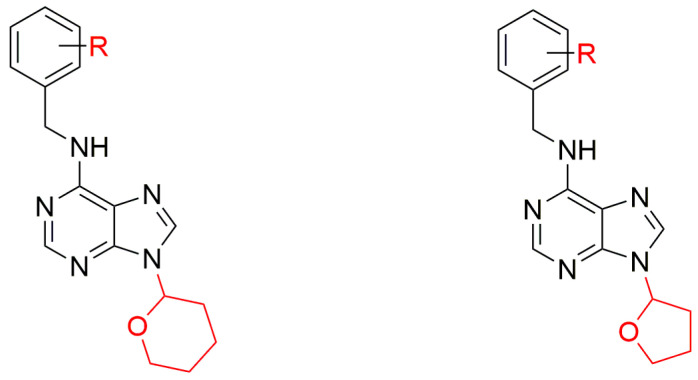
General structures of 9THP and 9THF ArCK derivatives (R = OH, OCH_3_ or their combination) [147].

In attempt to improve specific biological properties of CKs reported in 2009, a number of new hydroxyl and/or methoxy benzene ring-substituted 9THP and 9THF CKs (Figure 14) were synthesized and tested [147]. They were prepared via the condensation of 6-chloropurine with 3,4-dihydro-2H-pyran or 2,3-dihydrofuran, catalyzed by trifluoroacetic acid, followed by coupling of the intermediates with corresponding benzylamines [147].

The 9THP and 9THF ArCKs were found to have higher activities than corresponding free bases in tobacco callus, WLS, and *Amaranthus* bioassays. Not all the prepared 9THP and 9THF derivatives are entirely stable at pH < 4, because they slowly decompose to their free bases [147].

In 2012, 6-(3-methoxybenzylamino)-9-(tetrahydropyran-2-yl)purine (3MeOBAP9THP) derivative showed that it does not inhibit the primary root growth compared to the parent CK 6-(3-methoxybenzylamino)purine (3MeOBAP) [41]. Besides, the compound showed a positive impact on the growth of the aerial part compared to free base, all in the nanomolar (8 to 40 nM) concentration range [41]. This may be due to stimulation of ethylene biosynthesis, which correlated with observed root phenotypes and the strength of inhibition of root cell elongation. Root inhibition is probably caused by the formation of 9-glucosides, as explained above. An appropriate substituent at the purine N9 atom blocks its fast glucosylation and can thus protect the active CK from deactivation and prevent the primary root inhibition. Metabolic analysis with radioactively labelled 3MeOBAP9THP has revealed that the THP group can be slowly eliminated in vivo and its application indeed results in a significantly lower total content of inactive glucosides than treatments with unprotected 3MeoBAP [41]. In a study published the following year, the effects of 3MeOBAP9THP, 6-(3-methoxybenzylamino)-9-(tetrahydrofuran-2-yl)purine (3MeOBAP9THF), and 3MeOBAP on root elongation were compared [48]. 6-(3-methoxybenzylamino)-9-(tetrahydrofuran-2-yl)purine had a much weaker inhibitory effect than 3MeOBAP, but its ability to compete with tritium-labelled *tZ* for the activation site of the *A. thaliana* CRE1/AHK4 receptor in competitive receptor tests was comparable to that of 3MeOBAP [48]. Subsequently, physiological effects of these compounds have been tested in a number of micropropagation protocols, inter alia for horticultural and medicinal plants [167,168,169]. The results of their testing showed effects in diverse processes, e.g. acclimatization of micropropagated ‘Williams’ banana [170,171]. It indicates that these second-generation ArCKs have great potential for improving in vitro regeneration techniques for various economically important and endangered plants.

Recently, a large collection of 8-substituted 9THP CK derivatives was also synthesized [172]. Most were generated via multiple synthesis following previously published protocols [173,174,175] and substitution of the halogen atom at position C8 with a nucleophile (amine or alcoholate) to afford 8-substituted-9-THP-CK. The CK activity of all the compounds was determined in classical CK bioassays In the WLS assay, prepared compounds with a THP group generally had lower CK activity than the parent compounds. However, 8-chloro-9THP-iP and 8-bromo-9THP-iP exhibited very high activity over a wide concentration range, from 0.1 to 100 µM, in the tobacco callus bioassay (8–15% stronger activity than 1 µM BAP). Furthermore, all 8-substituted CKs with a 9THP group had comparable activities to their parent compounds (*t*Z, iP, and BAP) [172].

In view of the above structure-activity relationships resulting from the synthesis and testing of various 9-substituted ArCKs, new 9-substituted Kin derivatives were prepared and their antisenescence activity in WLS assays was investigated [176]. Seven Kin derivatives and analogues were prepared via nucleophilic substitution of 6-chloro-9-(tetrahydrofuran-2-yl)purine or 2,6-dichloro-9-(tetrahydrofuran-2-yl)purine with the appropriate amines. The most potent derivatives had slightly higher activity than BAP in WLS bioassays, similar to that of a previously synthesized compound, 6-furfurylamino-9-(tetrahydrofuran-2-yl)purine (Kin9THF).

Tetrahydropyranylation/tetrafuranylation of known CKs started a new era in the implementation of novel class of CK derivatives in tissue culture—their potential lies mainly in a small design change—a suitable easily removable substitution on N9 atom of purine, which prevents the formation of unwanted 9-glucoside associated with root inhibition. Besides, selected known CKs or newly developed mimetics of BAP and Kin substituted by these THP or THF groups retain very special antisenescent properties of CKs. 

In Table 1, we list derivatives with such combinations of N9, N6, C2 and C8 substitutions that were significantly more active than the widely applied classical CK compounds BAP, Kin and iP in three basic CK bioassays in the last 15 years.

## 4. Conclusions

The objective of this review was to describe, as far as possible, the endogenous occurrence, synthesis, and biological activity of numerous sugar and non-sugar 9-substituted CK derivatives. We have also covered their natural occurrence in plants in relation to their biological properties, toxicity and effects on plant growth and development, especially root and shoot development. We have summarized knowledge regarding natural disaccharide conjugates that are soluble in water, and thus particularly attractive for use in tissue culture. We have also summarized historical progress in their discovery and synthesis of these derivatives and highlighted several structural aspects of 9-substituted CKs and CK-like compounds, as well as their relationships to biological activities. Active derivatives and conjugates are summarized in Table 1, together with references. 

We analyzed the inactivation (reversible and reversible) of ArCKs and IsCKs through the formation of various forms of ribosides and glucosides and discussed the effect of their isomerism on CK activity. The discovery of new 9-substituted CKs and their potent developmental effects on plants has induced a boom in synthesis and testing of 9-substituted CK derivatives and their analogues useful in plant and human biotechnologies. Recently, the generation and testing of a number of new compounds has provided unexpected information on the biological properties of various 9-substituted CKs, whose research has historically been halted due to early conclusions by scientists about 9-glucoside inactivity in plants, since these compounds were considered to be the metabolic end-products of functional CKs. The development of new CK derivatives with knowledge of efficient structural motifs allows for an increase in their biological activities and thus provides interesting new molecules with various potential effects and metabolic advantages.

## Figures and Tables

**Table 1:** Summary of substitutions at N9, N6, C2, and C8 atoms, and their combinations, that resulted in compounds with significantly higher activity than appropriate standards in indicated CK bioassays.

Bioassay	Std.	Position of the Substituent on the Purine Ring				Ref.
		N9	N6	C2	C8	
***Amaranthus caudatus* betacyanin**	**BAP**	β-D-ribofuranosyl-	2-chlorobenzyl-, 3-chlorobenzyl-, 2-bromobenzyl-, 3-bromobenzyl-, 3-iodobenzyl-, 3,5-difluorobenzyl-, 2,4,5-trifluorobenzyl-, 2-chloro-4-fluorobenzyl-, 2-trifluoromethylbenzyl-, 3-trifluoromethoxybenzyl-	H	H	[45]
			2-fluorobenzyl-, 3-fluorobenzyl-, 4-fluorobenzyl-, 2-chlorobenzyl-, 3-chlorobenzyl-, 2-bromobenzyl-, 3-bromobenzyl-, 4-bromobenzyl-, 3-iodobenzyl-	Cl	H	[46]
		2′-deoxy-β-D-ribofuranosyl-	3-hydroxybenzyl-, 2-fluorobenzyl-, 4-fluorobenzyl-, 2-chlorobenzyl, 3-chlorobenzyl-, 2-bromobenzyl-, 3-brombenzyl-, 2-methybenzyl-, 2-trifluoromethylbenzyl-	H	H	[120]
		tetrahydropyran-2-yl	isopentenyl-, furfuryl-	H	3-aminopropyl-, 4-aminobutyl-, methylsulfanyl-, dimethyl-, allyl-	[172]
			benzyl-	H	H	[147]
		tetrahydrofuran-2-yl	benzyl-, 2-methoxybenzyl-, 3-methoxybenzyl-	H	H	[147]
			thiofen-2-yl-	Cl	H	[176]
	**iP**	tetrahydropyran-2-yl, ethoxyethyl-, 2-bromoethyl-, 2-chloroethyl-, 4-ethoxy-4-oxobutyl-	isopentenyl-	H	H	[158]
**Senescence (WLS)**	**BAP**	β-D-ribofuranosyl-	2-fluorobenzyl-, 3-fluorobenzyl-, 4-fluorobenzyl-, 2-chlorobenzyl-, 4-chlorobenzyl-, 2-methylbenzyl-, 3-methybenzyl-, 2-methoxybenzyl-, 3-methoxybenzyl-, 3,4-dichlorobenzyl-, 2,3-dimethoxybenzyl-, 2,4-difluorobenzyl-, 3,5-difluorobenzyl-, 2,3,4-trifluorobenzyl-, 2,3,6-trifluorobenzyl-, 2-chloro-4-fluorobenzyl-, 3-chloro-4-fluorobenzyl-, 2-hydroxy-5-methylbenzyl-, 2-difluoromethoxybenzyl-	H	H	[45]
			2-fluorobenzyl-, 3-fluorobenzyl-, 4-fluorobenzyl-, 2-chlorobenzyl-, 3-chlorobenzyl-,4-chlorobenzyl-, 3-bromobenzyl-, 4-bromobenzyl-	Cl	H	[46]
		β-D-arabinofuranosyl-	benzyl-, 2-fluorobenzyl-, 3-fluorobenzyl-, 4-fluorobenzyl-, 3-chlorobenzyl-, 2-methoxybenzyl-,3-methoxybenzyl-, 3-hydroxybenzyl-, 3-methylbenzyl-, 2,5-difluorobenzyl-, 3,5-difluorobenzyl-	H	H	[134]
		2′-deoxy-β-D-ribofuranosyl-	benzyl-, 2-hydroxybenzyl-, 3-hydroxybenzyl-, 4-hydroxybenzyl, 3-methoxybenzyl-, furfuryl- 2,5-dimethoxybenzyl-, 2-fluorobenzyl-, 3-fluorobenzyl-, 4-fluorobenzyl-, 2-chlorobenzyl-, 3-chlorobenzyl-, 4-chlorobenzyl-, 2-bromobenzyl-, 3-bromobenzyl-, 4-bromobenzyl-, 2-methylbenzyl-, 3-methylbenzyl-, 2-trifluoromethylbenzyl-, 3-trifluoromethylbenzyl-,	H	H	[120]
		tetrahydropyran-2-yl	benzyl-, 3-hydroxybenzyl-, 2-methoxybenzyl-,	H	H	[147]
		tetrahydrofuran-2-yl	benzyl-, 3-hydroxybenzyl-	H	H	[147]
			tetrahydrofuran-2-yl-, thiofen-2-yl-, 5-methylthiofen-2-yl-	H	H	[176]
			tetrahydrofurfuryl-	Cl	H	[176]
	**Kin**	2-bromoethyl-, 2-chloroethyl-, 4-chlorobutyl-, 1-ethoxyethyl-, tetrahydrofuran-2-yl	furfuryl-	H	H	[157]
**Tobaccocallus**	**BAP**	β-D-ribofuranosyl-	2-fluorobenzyl-, 4-fluorobenzyl-, 2-bromobenzyl-, 2-methoxybenzyl-	H	H	[45]
			2-flourobenzyl-, 3-fluorobenzyl-, 4-fluorobenzyl-, 2-chlorobenzyl-, 3-chlorobenzyl-, 4-bromobenzyl-	Cl	H	[46]
		2′-deoxy-β-D-ribofuranosyl-	benzyl-, 4-fluorobenzyl-, furfuryl-	H	H	[120]
		tetrahydropyran-2-yl	isopentenyl-, furfuryl-	H	2-aminoethyl-, 3-aminopropyl-, 4-aminobutyl-, 6-aminohexyl-, methoxy-, 2-hydroxyethyl-	[172]
			benzyl-	H	H	[147]
		tetrahydrofuran-2-yl	furfuryl-, thiofen-2-yl, 5-hydroxymethylfuran-2-yl-	H	H	[176]
			furfuryl-, tetrahydrofurfuryl-, thiofen-2-yl-	Cl	H	[176]
	**iP**	ethoxyethyl-, acetoxy-, 2-azidoethyl-, 4-chlorobutyl-, 3-cyanopropyl-	isopentenyl-	H	H	[158]
	**Kin**	2-bromoethyl, 2-chloroethyl-, 1-ethoxyethyl-, tetrahydrofuran-2-yl	furfuryl-	H	H	[157]
